# ERGA-CBP chromosome-level genome assembly of the blind scorpion *Belisarius xambeui* Simon, 1879 (Belisariidae, Scorpiones), a singular scorpion in Europe

**DOI:** 10.12688/openreseurope.22872.2

**Published:** 2026-04-21

**Authors:** Sara Guirao-Rico, Vadim A. Pisarenco, Paula Escuer, Pau Balart-García, Marc Domènech, Adrià Bellvert, Silvia Adrián-Serrano, Alejandro Sánchez-Gracia, Miquel A. Arnedo, Julio Rozas

**Affiliations:** 1 Universitat de Barcelona Departament de Genetica Microbiologia i Estadistica, Barcelona, Catalonia, 08028, Spain; 2 Universitat de Barcelona Institut de Recerca de la Biodiversitat, Barcelona, Catalonia, 08028, Spain; 3 Universite de Neuchatel Institut de Biologie, Neuchâtel, Canton of Neuchâtel, Switzerland; 4 Fish Evolution Research Group, Department of Zoology, Faculty of Science, Charles University, Prague, Czech Republic; 5 Universitat Greifswald Zoologisches Institut und Museum, Greifswald, Mecklenburg-Vorpommern, Germany; 6 Departament de Biologia Evolutiva Ecologia i Ciencies Ambientals, Universitat de Barcelona, Barcelona, Spain

**Keywords:** Long-read sequencing, reference genome, scorpion, European Reference Genome Atlas, Biodiversity Genomics Europe, Earth Biogenome

## Abstract

We present a chromosome-level reference genome for the blind scorpion *Belisarius xambeui* Simon, 1879 (Belisariidae, Scorpiones). The genome size estimated by flow cytometry (4.32 Gb) closely matches the final assembly size (3.98 Gb). The final assembly comprises 19,045 scaffolds, including 56 chromosome-level scaffolds (pseudochromosomes) that account for 90.08% of the total assembly. Nucleotide diversity across the genome was low, with an average *π* of 0.0018.

## Background

**Figure 1.**
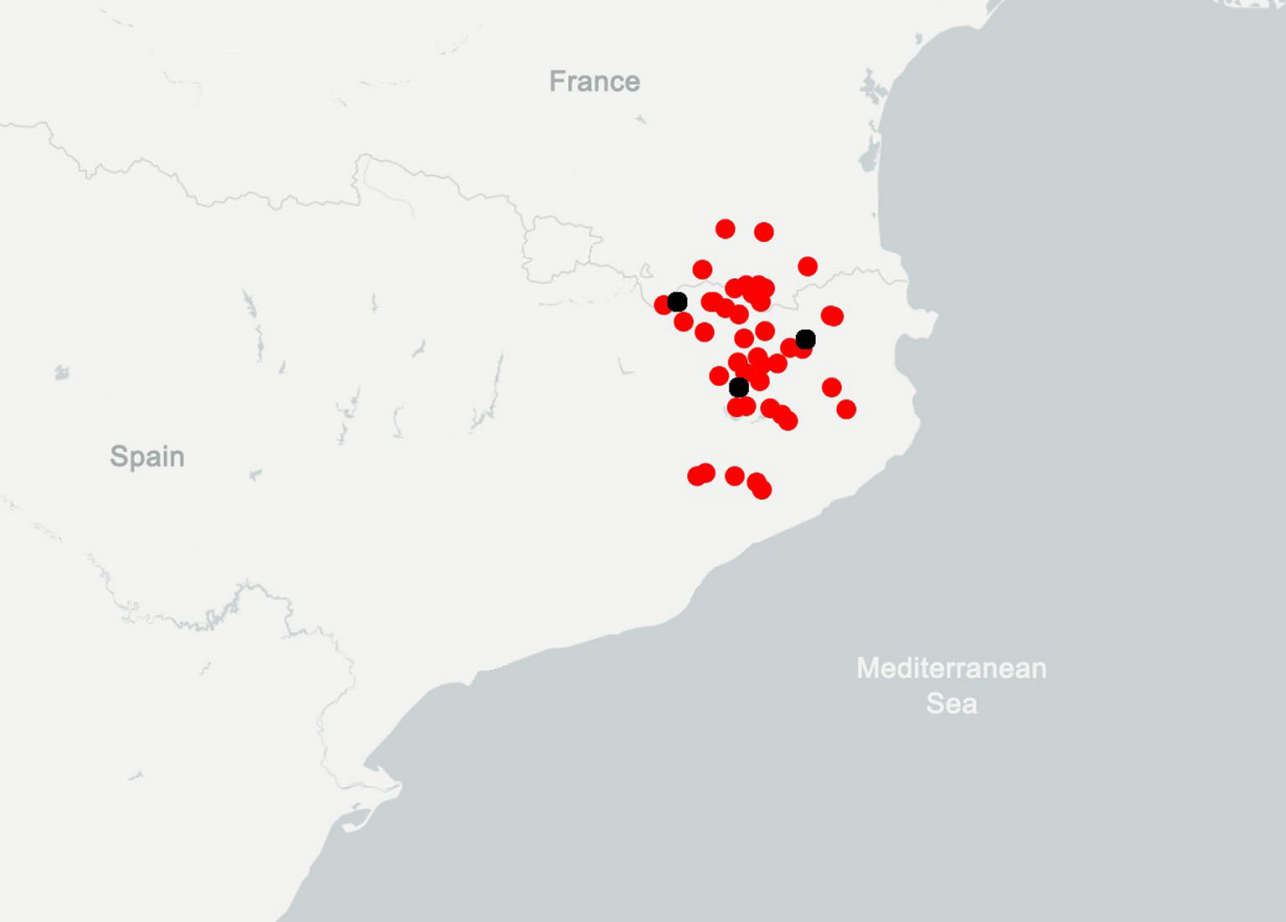
Distribution of *B. xambeui.* Modified from GBIF.org, it includes *B. xambeui* sampling sites (black points) and records from museums, zoological collections and citizen science platforms (red points).

**Figure 2.**
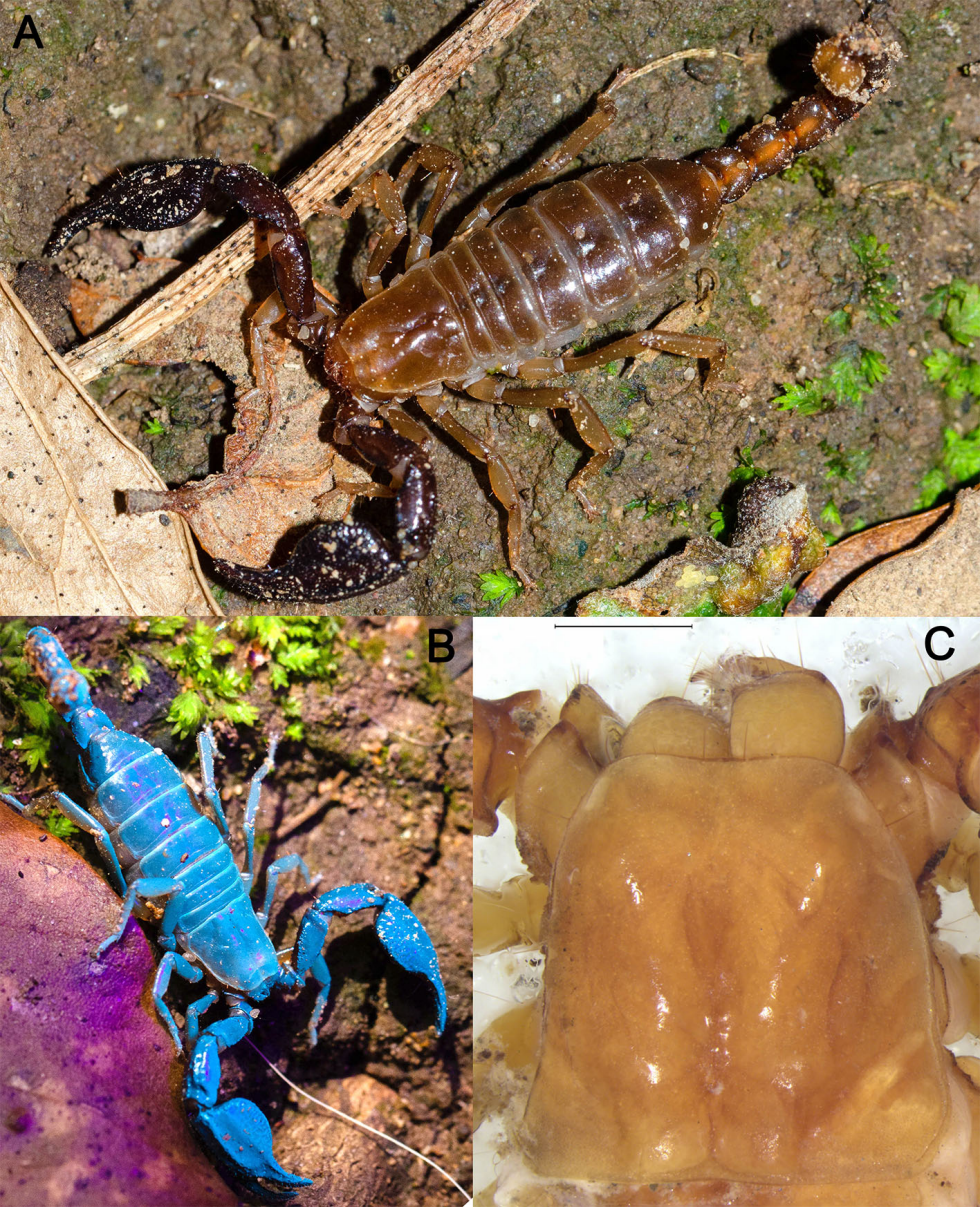
A) An adult specimen of *B. xambeui* in its habitat, in Osona (Catalonia). B) A specimen of *B. xambeui* glowing blue under black light (UV-A). Compounds in the scorpion’s cuticle absorb ultraviolet light and re-emit it as visible fluorescence. C) The eyeless carapace of a young specimen under the stereomicroscope; scale bar: 1 mm.

Scorpions represent an iconic lineage of arthropods, long recognized for their unique body plan, ancient fossil record, and venom potency. The blind scorpion *Belisarius xambeui* Simon 1879 is a small species (~3 cm) that constitutes a narrow Catalan endemism ( Simon, 1879; Lourenço, 2015). It is distributed in the eastern Pyrenees and Pre-Pyrenees and northernmost part of the Catalan Pre-Costal mountain range ( Figure 1). It is usually found under leaf litter and buried stones in humid and shady habitats, as well as in caves. Its morphology is highly modified, characterized by the absence of eyes, and the extreme reduction in the number of pectinal teeth (only 4–6), which serve as the chemosensory organ ( Figure 2). This species was the first troglomorphic scorpion ever described, and it is considered one of the most cryptic in Europe, with very small and highly threatened populations ( Vignoli *et al.*, 2008). Moreover, its low fertility (5–24 eggs per clutch, Auber, 1959) may further compromise its long-term survival. Indeed, the species is listed as threatened with extinction in the Catalan catalog of threatened wild fauna ( https://portaldogc.gencat.cat/utilsEADOP/PDF/8758/1927723.pdf).

The taxonomic position of *B. xambeui* remains uncertain. It was initially allied with the family Troglotayosicidae, which comprises a single genus of humicolous and cave-dwelling species native to Ecuador and Colombia, in South America. More recently, however, the family Belisariidae originally described by Lourenço (1998) has been resurrected to accommodate the blind scorpion along with a recently described cave-dwelling species from southern Iberian Peninsula ( *B. ibericus* Lourenço, 2015), as well as the newly established genus and species *Sardoscorpius troglophilus* Tropea & Onnis, 2020, found in caves in Sardinia ( Tropea & Onnis, 2020).

This new genome, distantly related to the species with previously available genomic data, will provide new insights into the phylogenetic position of *B. xambeui* within the scorpions tree of life. At present, genomic data are available for only seven scorpion species, namely the hadrurid *Hadrurus arizonensis* Ewing, 1928, the buthids *Olivierus martensii* (Karsch, 1879) (= *Mesobuthus martensii*), *Olivierus przewalskii* (= *Mesobuthus przewalskii*) (Birula, 1897), *Androctonus mauritanicus* (Pocock, 1902), *Centruroides vittatus* (Say, 1821), *Centruroides sculpturatus* Ewing, 1928, and the euscorpiid *Euscorpius feti* Tropea, 2013 ( Cao *et al.*, 2013; Schwager *et al.*, 2017; El Ghoubali *et al.*, 2022; Bryant *et al.*, 2024; Yamashita *et al.*, 2024; Su *et al.*, 2025; National Center for Biotechnology Information (NCBI) genome dataset, https://www.ncbi.nlm.nih.gov/datasets/genome/). Scorpions comprise two major clades: Buthida, which includes buthids and other families with generally stronger venoms, and Iurida, which includes belisariids, hadrurids and euscorpiids, among others, commonly with less potent venoms. Recent estimates suggest that these two main lineages diverged approximately 291–320 million years ago ( Santibáñez-López *et al.*, 2022). The new genome of *B. xambeui* will also yield valuable information on the genomic basis of fundamental biological traits, such as changes in the venom composition and sensory perception (i.e., vision and chemosensation) ( Garb *et al.*, 2018). More broadly, the inclusion of this genome will substantially enhance the currently limited genomic resources available for non-insect arthropods. Despite the remarkable diversity and ecological relevance of chelicerates (about 100,000 species), less than 100 species have been sequenced at the chromosome level. Within the Scorpiones order (about 2,500 species), the *Belisarius* genome represents the third high-quality reference genome. It thus constitutes an important resource for studying chelicerate genome structure and evolution, offering a valuable genome for the broader scientific community.

## Material and methods

### Sample collection, processing and genome size estimation

Three specimens of *B. xambeui* ( Figure 2) were collected in three different locations in Catalonia (Spain) and in three different years. One individual, sampled in 2019 in the Cova de la Mosquera (Beuda, Girona), was used for the PacBio genome sequencing, and whole-genome Illumina resequencing. The second individual, sampled in 2020 in Cantonigròs, was used for flow cytometry. The third one, sampled in 2021 in Queralbs, was used to generate the Hi-C libraries. Because specimens of *B. xambeui* are rare, and in order to secure the highest quality of DNA, we used specimens in the best condition of preservation, which motivated the decision to use specimens from different localities. Sampling was conducted with authorization from the Generalitat de Catalunya’s Departament de Territori i Sostenibilitat, and the help of the Federació Catalana d’Espeleologia. All individuals except the one used for flow cytometry were preserved either in ethanol 100% at -20ºC or flash frozen at -80ºC (Table S1). We estimated the genome size from a single adult individual (Table S1) using flow cytometry as in Sánchez-Herrero *et al.* (2019). *B. xambeui* specimens are easily identifiable by the absence of eyes and the reduced number of teeth on the pectines, which clearly distinguish them from the other two scorpion species inhabiting the region, *Buthus occitanus* (Pierre-Joseph Amoreux, 1789) and *Tetratrichobothrius flavicaudis* De Geer, 1778. Furthermore, the species identity of all individuals was confirmed in the laboratory using the mitochondrial cytochrome c oxidase subunit I (COI) as DNA barcode prior to DNA sequencing. The sex of the sequenced individuals is unknown, because a careful examination under the microscope was not possible since the specimens needed to remain frozen in order to ensure correct preservation for genome sequencing. Vouchers are deposited at the Centre de Recursos de Biodiversitat Animal of the Universitat de Barcelona (codes MZB2026-0200 and MZB2026-0201).

### DNA extraction

We extracted a high molecular weight genomic DNA (HMW gDNA) from a single flash-frozen individual using the Blood & Cell Culture DNA Mini Kit (Quiagen) for both long and short read sequencing (Table S2). We measured the DNA extraction quality using a NanoDrop spectrophotometer, yielding absorbance ratios of 260/280 = 1.74 and 260/230 = 2.26. We built Arima Hi-C libraries at the Genomics Unit of the Institute of Evolutionary Biology (Spain; https://www.ibe.upf-csic.es/genomics-unit ) following the manufacturer’s protocol (Arima Genomics) using a single, flash-frozen whole specimen (Table S2).

### Genome sequencing

HMW gDNA was sequenced using PacBio CLR technology, which resulted in average coverage of 84x complemented by short-read sequencing on the Illumina NovaSeq 6000 platform (150PE; ~27x of coverage), both carried out at Novogene. For the scaffolding process, the Arima Hi-C libraries were sequenced using Illumina NovaSeq 6000 platform (150PE; 66x of coverage) at the Centre Nacional d’Anàlisi Genòmica (CNAG) sequencing unit (Spain) (Table S2).

### Genome assembly and manual curation

*De novo* genome assembly was performed in two steps. First, PacBio reads were converted to FASTA format using the commands bam2fq from samtools v.1.9 and seq -A from seqtk v.1.3-r117-dirty. The resulting FASTA reads were assembled with wtdbg2 v.2.5 ( Ruan & Li, 2020). To ensure optimal assembly quality, we generated six independent assemblies, differing in three key parameters of wtdbg2: minimum read length ( -L), minimum alignment length ( -l), and enabling or not the realignment mode ( -R) (Table S3a). PacBio reads were aligned back to each assembly with minimap v.2.22 ( Li, 2018) for polishing. Assembly statistics, including the number of contigs, mean and the median of the contig length, and N50, were computed using the script seq_n50.pl (provided by wtdbg2 software) (Table S3b). Assembly completeness was determined using BUSCO-v2.5.4 ( Seppey *et al.*, 2019), with the odb10 databases (Arachnida; 2,934 genes; Arthropoda; 1,013 genes and Eukaryota; 255 genes) ( Kriventseva *et al.*, 2019).

Potential haplotype duplications (i. e., haplotigs and contig overlaps) were evaluated with purge_dups v1.2.6 ( Guan *et al.*, 2020), with default parameters and the cutoffs rechecked - m 48 -u 288. minimap v.2.22 ( Li, 2018) was also used within the purge_dups pipeline. To assess and remove putative organismal contaminations, we used the BlobTools2 from BlobTools kit pipeline ( Kumar *et al.*, 2013; Laetsch & Blaxter, 2017), using the NCBI nucleotide and UniProt databases (updated in July 2021 and June 2021, respectively) ( https://www.ncbi.nlm.nih.gov/nucleotide/, The UniProt Consortium, 2021), along with the aforementioned BUSCO odb10 databases.

To further improve the quality of the assembly, we addressed potential mitochondrial contamination. Putative mitochondrial contigs were identified to enable their removal or masking from the nuclear genome assembly. Specifically, all assembled contigs were queried using BLAST against two reference datasets: i) the *B. xambeui* mitochondrial assembly, and ii) a set of 11 complete mitochondrial genomes from 10 different scorpion species (Table S4). Only BLAST hits with an E-value ≤ 1×10 ^-50^ were considered significant and thus subject to masked or removal. Hits shorter than 1,000 bp were disregarded. For contigs exhibiting significant mitochondrial hits, a conditional masking strategy was applied: i) if the non-mitochondrial portion of the contig exceeded 3,000 bp, the identified mitochondrial segment was excised and replaced with 15-base ‘N’ placeholder sequence to preserve the putative nuclear portion of the contig; ii) if the remaining non-mitochondrial portion of the contig was 3,000 bp or less, the entire contig was discarded from the assembly. This criterion ensured the retention of high-confidence nuclear contigs while removing sequences likely to be mitochondrial or too short for reliable assembly.

We obtained a chromosome-level assembly by scaffolding the draft genome using the Arima Hi-C data. First, raw Hi-C reads were preprocessed and quality-trimmed using fastp with default parameters ( Chen *et al.*, 2018). Second, trimmed paired Hi-C reads were mapped against the draft assembly using bwa mem-v0.7.17-r1198-dirty ( Li & Durbin, 2009), designating the 5’ coordinate as the primary alignment and skipping rescuing mates and read pairing ( -5SP). At this step, the minimum mapping quality was set to 0 ( -T0). Third, high-quality ligation events (Hi-C pairs) were identified from the resulting SAM file using the pairtools parse command from Pairtools-v1.1.2 ( Open2C *et al.*, 2024). In this step, reads with a minimal mapping score below 30 were considered as multi-mapping ( -min-mapq 30). In addition, we filtered out complex walks (multiple ligation events), retaining only the 5’-most unique alignment on each side ( -walks-policy 5unique), and ignoring gaps of up 30 bp between alignments ( -max-inter-align-gap 30). Fourth, the resulting pairsam file was sorted with (“pairtools sort”), PCR duplicates were removed (“pairtools dedup”), and the final BAM files were generated (“pairtools split”). Finally, the wtdbg2 draft genome assembly and the processed Hi-C BAM file were used as input for YaHS-v1.2.2 ( Zhou *et al.*, 2023), run with default parameters, to generate the chromosome-scale scaffolds. The resulting assembly was visually inspected and manually curated by generating the Hi-C contact map using JuicerTools-v1.2.2 ( Durand *et al.*, 2016b) and examined them in JuiceBox-v1.11.08 ( Durand *et al.*, 2016a). This inspection enabled the identification of potential misjoints and misplaced scaffolds. Manual curation resolved two misjoins and corrected a few incorrectly placed scaffolds.

### Repetitive elements analysis

We used RepeatModeler-v2.0.6 ( Flynn *et al.*, 2020) for *de novo* identification of repetitive elements (REs), using the LTR discovery pipeline ( -LTRStruct). Sequences classified as “unknown” were subsequently reclassified with DeepTE ( Yan *et al.*, 2020) using the metazoan model ( -sp M). The resulting RE libraries were merged into a single comprehensive database, which was then used as input for masking with RepeatMasker-v4.1.7 ( http://repeatmasker.org), configured with Dfam 3.8 ( Storer *et al.*, 2021), an executed using the sensitive search mode ( -s). Transposable elements (TEs) identified by RepeatMasker were further classified using custom Python scripts based on their class, subclass, order and superfamily, following the hierarchical classification of Wicker *et al.* (2007) and Wells & Feschotte (2020).

### Nucleotide diversity

We estimated the per-site nucleotide diversity ( *π*) with ANGSD v0.940-dirty ( Korneliussen *et al.*, 2014), using Illumina resequencing data from a single individual, sequenced at an average coverage depth of ~27X. Firstly, we assessed the quality of raw reads using FASTQC v0.11.9 (Andrews (2010), http://www.bioinformatics.babraham.ac.uk/projects/fastqc/). Reads were trimmed with Trimmomatic v0.39 ( Bolger *et al.*, 2014), to filter out adapters, reads shorter than 50bp ( MINLEN:50), and low-quality bases using a sliding window of 4bp with an average Phred score of 15 ( SLIDINGWINDOW:4:15). We also removed leading and trailing bases with low quality scores (<3) ( LEADING:3, TRAILING:3), as well as all missing bases. Secondly, filtered reads were mapped against the reference genome of *B. xambeui* using bwa mem v0.7.16 ( Li & Durbin, 2009). Next, the generated SAM file was converted to BAM format, sorted and indexed using samtools v1.21 ( Danecek *et al.*, 2021). Thirdly, we estimated the sample allele frequency (SAF) likelihoods with ANGSD using the BAM file as input. We applied specific quality filters to filter out positions with very low or very high coverage ( -setMinDepth 10, -setMaxDepth 90). In addition, we only retained positions with high base calling quality, properly paired and high mapping quality reads ( -minQ 20 -only_proper_pairs -minMapQ 20). We set the mapQ parameter for excessive mismatches ( -C 50) and the qscores around indels ( -baq 1). We discarded reads that did not map uniquely ( -uniqueOnly 1), as well as those not primary, failure and duplicate reads ( -remove_bads 1). We set the *B. xambeui* reference genome as both the reference ( -ref) and the ancestral ( -anc) sequence, and estimated the genotype likelihoods per-site using the GATK algorithm ( -GL 2) assuming Hardy-Weinberg Equilibrium ( -doSaf 1). In addition, we used the -doCounts 1 option to count the bases per site after applying the abovementioned depth and base quality filters. The resulting SAF file was used as input for the realSFS program to estimate the folded site frequency spectrum (SFS) ( -fold 1), and to calculate the thetas per site (realSFS saf2theta). Finally, we retrieved the nucleotide diversity ( *π*; Nei, 1987) for each chromosome using the thetaStat program (thetaStat do_stat) within ANGSD (see also ( Kousathanas *et al.*, 2017)).

### Mitochondrial assembly and annotation

To assemble the mitogenome of *B. xambeui*, we first performed a BLAST using a mtDNA sequence of the scorpion *Centruroides vittatus* (GenBank ID: MF975702.1) to retrieve all publicly available complete scorpion mitochondrial genomes, resulting in a dataset of 11 complete mtDNA sequences (Table S4). These genomes were subsequently used as queries in a BLAST search against PacBio long-read dataset from *B. xambeui* to identify mitochondrial reads, applying an E-value threshold of < 1e-4. All reads with significant matches against these scorpion mitogenomes were subsequently assembled *de novo* using wtdbg2 v.2.5 ( Ruan & Li, 2020), and the resulting assembly was polished using PacBio long reads. Mitochondrial genome annotation was performed using MITOS2 ( Bernt *et al.*, 2013, available at http://mitos2.bioinf.uni-leipzig.de/index.py), with default parameters, to infer protein-coding genes (PCGs) and ribosomal RNAs (rRNAs). Transfer RNAs (tRNAs) were annotated using both MITOS2 and ARAGORN ( Laslett & Canback, 2004), allowing inference of their primary sequences and secondary structures. Gene boundaries were subsequently manually curated based on alignments between the mitochondrial genome of species *B. xambeui* and eight scorpion mitogenomes (Table S5) retrieved from public databases.

## Results

### Nuclear genome sequence report

Flow cytometry analysis estimated the *B. xambeui* genome size at 4.32 Gb, approximately four times larger than those reported for other scorpion species (0.88 Gb–1.32 Gb) (Gregory (2026), http://www.genomesize.com), and nearly two times than those estimated in *Hadrurus arizonensis* based on the total assembly size ( Bryant *et al.*, 2024), and similar to that reported for *Euscorpius feti* (4.4 Gb) (National Center for Biotechnology Information (NCBI) genome dataset, https://www.ncbi.nlm.nih.gov/datasets/genome/). The optimal assembly (Table S3b), selected based on its superior contiguity and completeness, comprised 24,679 contigs, 373 of which were identified as potential contaminants (assigned to Proteobacteria and other non-target taxa, based on BLAST and Diamond hits) and were excluded. We also identified 96 contigs with significant mitochondrial BLAST hits (E-value < 1e-50), 7 were completely removed, 21 were masked and 89 were disregarded since they did not pass the filter of the BLAST E-value or the total length that match mtDNA was below 1,000 bp. The resulting *B. xambeui* genome assembly was ~3.97 Gb, including 24,299 contigs, with a contig N50 ~1.07 Mb. This assembly had a high completeness, with 91.4%, 89.8% and 87.9% of complete BUSCO genes identified across the Arachnida, Arthropoda and Eukaryota datasets, respectively. After the assembly and polishing step, we used BlobTools2 from BlobTools kit ( Kumar *et al.*, 2013; Laetsch & Blaxter, 2017) to assess the assembly quality, identify and remove potential contaminant scaffolds.

**Figure 3.**
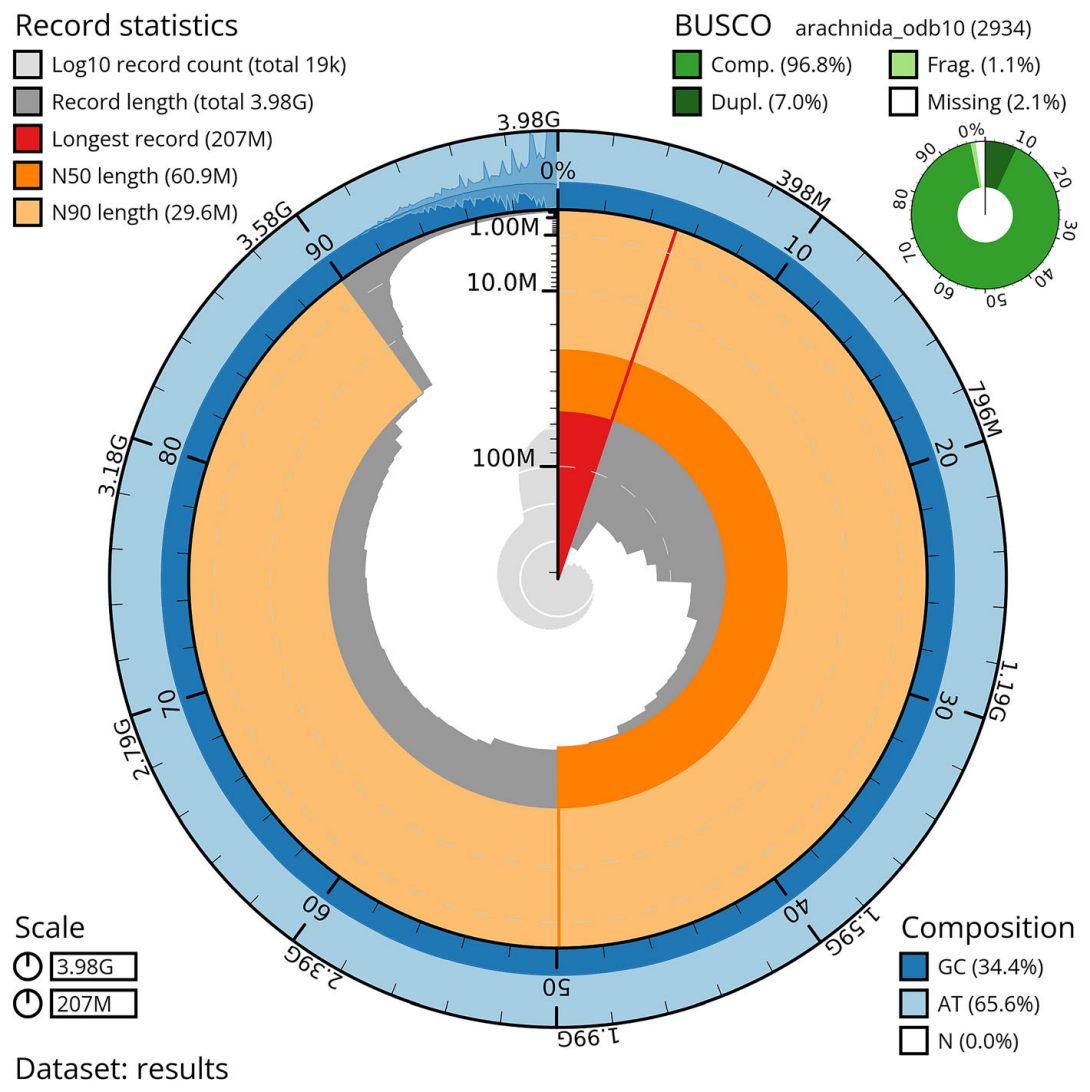
Snail plot summary of genome assembly of *B. xambeui.* The BlobToolKit snail plot shows N50 metrics and BUSCO gene completeness. The main plot is divided into 1,000 bins around the circumference with each bin representing 0.1% of the 3,979,917,391 bp assembly. The distribution of sequence lengths is shown in dark grey, with the plot radius scaled to the longest sequence in the assembly (207,115,667 bp, shown in red). Orange and pale-orange arcs indicate the N50 and N90 sequence lengths (60,866,488 and 29,580,904 bp), respectively. The pale grey spiral shows the cumulative sequence count on a log scale, with white scale lines indicating successive orders of magnitude. The blue and pale-blue outer bands show the distribution of GC, AT and N percentages across the same bins as the inner plot. A summary of complete, fragmented, duplicated and missing BUSCO genes from the arachnida_odb10 dataset is shown in the top right.

**Figure 4.**
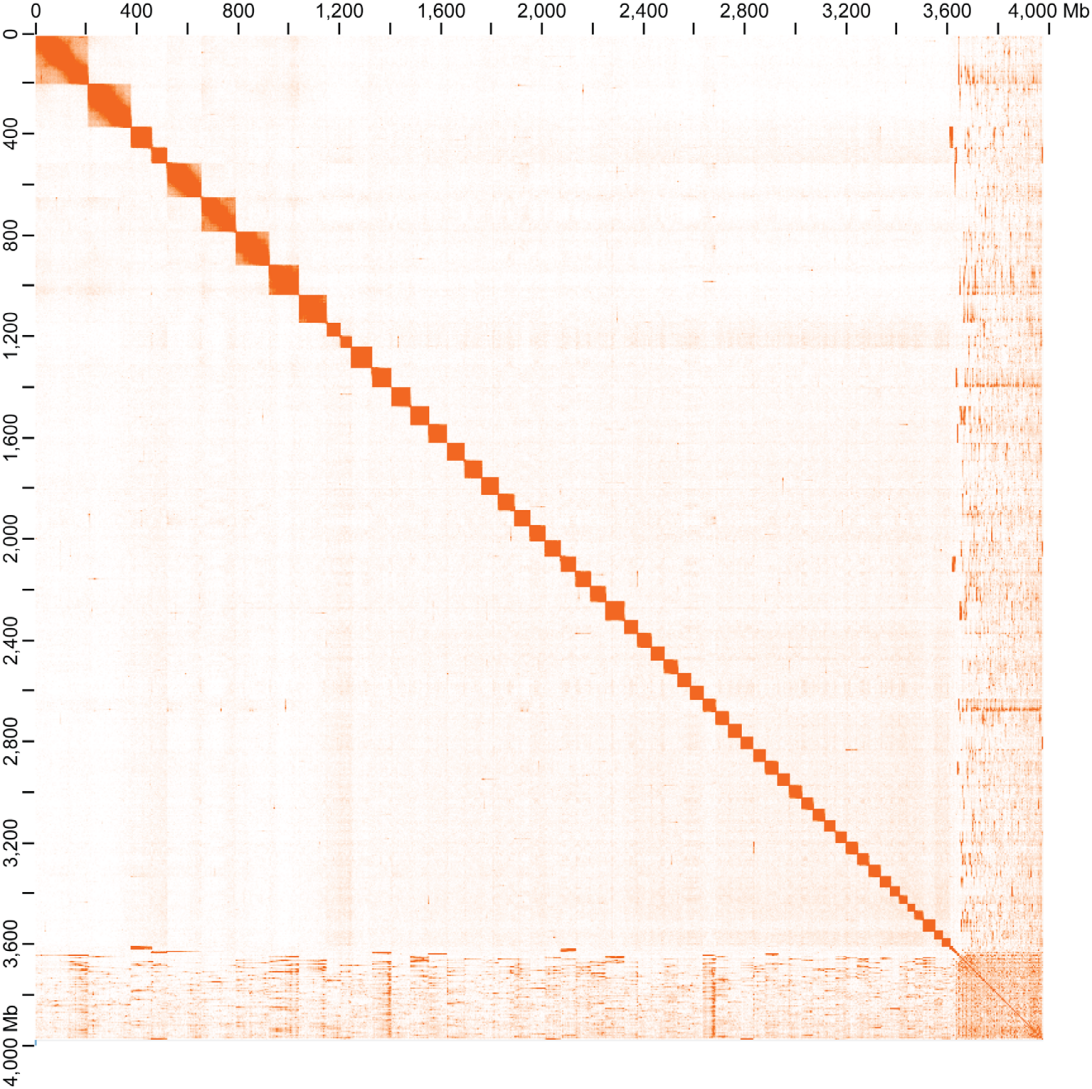
Hi-C contact map of the chromosome-level genome assembly of *B. xambeui* visualized using JuiceBox.

The scaffolded assembly comprised 19,045 scaffolds with a N50 of ~207 Mb, including approximately 56 chromosome-level scaffolds (pseudochromosomes) that contain 90.08% of the assembled sequences ( Figure 3, Figure 4, Table 1 and Table S6). Remarkably, the complete BUSCO scores increased to 96.8%, 96.5% and 97.3% across the Arachnida, Arthropoda and Eukaryota datasets, respectively.

**Table 1.** Genome assembly and annotation statistics.

Assembly level	Chromosome
Assembly size (Mb)	3,979.92
GC Content (%)	34.37%
Number of scaffolds	19,045
Longest scaffold (Mb)	207.1
Scaffold N50 (Mb) / L50	60.9 / 21
Scaffold N90 (Mb) / L90	29.6 / 55
Chromosomes ^a^ & Sequence in chromosomes (%)	56 / 90.08%
BUSCO completeness ^b^	
Genome	C:96.8% [S:89.8%,D:7.0%], F:1.1%,M:2.1%,n:2,934
Repetitive regions (%)	68.4%
Nucleotide diversity	0.0018

^a^ Haploid number of chromosomes.

^b^ BUSCO scores based on the arachnida_odb10 BUSCO set using version 5.4.2. C = complete [S = single copy, D = duplicated], F = fragmented, M = missing, n = number of orthologues in comparison.

## Mitogenome sequence report

The mitochondrial genome assembly of *B. xambeui* yielded an almost complete mitogenome of 14,529 bp, consistent with sizes reported for related scorpions. Assembly accuracy was validated by BLAST comparison against the available COI sequence of *B. xambeui* (GenBank ID: JN018156), showing near-identical sequence similarity. We found that the assembly was likely incomplete at the *nad5* gene, with a missing fragment of ~1,018 bp, that the *atp6* gene includes a deletion that disrupted the reading frame. Automatic annotation recovered all protein coding genes, both rRNAs, and all tRNAs except *trnK*, which was manually annotated by comparison with homologous sequences. Manual curation confirmed canonical secondary structures for all tRNAs and corrected minor issues in *trnF*, *trnL1*, *trnM* and *trnS1.* Among the 22 tRNAs, five ( *trnC*, *trnD*, *trnF*, *trnG*, *trnH*) lacked the T arm, *trnS1* lacked the D arm, whereas the remaining had full structures. *trnF* showed two possible secondary structures, and *trnR* exhibited an unusual “bubble” caused by an asymmetric mismatch in the acceptor stem. Notably, we identified a >600 bp insertion between *trnW* and *trnC*, which is absent from other scorpion mitogenomes, with similarity to the mitochondrial control region. We validated this issue by PCR, using primers designed at the coding regions of *nad2* and *cox1*, confirming that this insertion is not an assembly artifact, but likely represents a duplicated fragment of the control region that has been inserted between *nad2* and *cox1* in *B. xambeui.*

### Nucleotide diversity

We found that the levels of nucleotide diversity are low, with an average of *π* = 0.0018, relatively homogeneous across chromosomes (ranging from a maximum of *π* = 0.0028 and a minimum of *π* = 0.0011. The values are nearly identical when excluding repetitive regions (Table S7). Although these values indicate a somewhat reduced level of genetic variation, they are likely not low enough to raise immediate concern regarding a severe loss of polymorphism or compromised adaptive potential. However, since these estimates are derived from a single individual they should be interpreted with caution, as they may not fully capture population-level variation. Moreover, although nucleotide diversity could be an important criterion in conservation assessments, it should not be considered as the sole factor guiding scientific-based conservation recommendations. Although some studies have reported a relationship between nucleotide diversity and evolutionary responses, there is no simple general relationship between genetic diversity and the risk of species extinction ( Ørsted *et al.*, 2019; Teixeira & Huber, 2021; Jeon *et al.*, 2024).

## Data Availability

Genome sequencing data have been submitted to the European Nucleotide Archive (ENA) ( https://www.ebi.ac.uk/ena/browser/home) under the umbrella study PRJEB54934, including PRJEB54932 (raw sequencing data) and PRJEB54933 (chromosome-level genome assembly). The genome assembly is also accessible through NCBI (GCA_982267015.1). Genomic data includes PacBio CLR long-reads (ERR10089999, ERR10090000, ERR10090001), Illumina short-reads (ERR10088334, ERR10088335, ERR10088336, ERR10088337) and Arima Hi-C data (ERR16820444-16820459). Information of the DNA sample of the individual PB0001 has been deposited under the accession number ERS12764351. Additional information of the genome assembly is available at https://github.com/molevol-ub/Belisarius_xambeui.
