# Supplementary material for: ERGA-CBP chromosome-level genome assembly of the blind scorpion Belisarius xambeui Simon, 1879 (Belisariidae, Scorpiones), a singular scorpion in Europe
Source: Open Res Eur. 2026 Apr 21;6:53. Originally published 2026 Feb 17. [Version 2] doi: 10.12688/openreseurope.22872.2 (PMC12957889; doi:10.12688/openreseurope.22872.2)
Supplement: Peer review history

# Reviewer report: Reviewer response for version 2

**DOI:** 10.21956/openreseurope.25418.r72791

**Published:** 2026-04-23

**Authors:** Javier Blasco-Aróstegui

**Affiliations:** 1 Universidade de Lisboa, Lisbon, Lisbon, Portugal

The authors have appropriately addressed all the suggested edits and the manuscript is more than ready for indexing, comprising a fantastic contribution to scorpiology.

Are sufficient details of methods and materials provided to allow replication by others?

Partly

Is the rationale for creating the dataset(s) clearly described?

Yes

Are the datasets clearly presented in a useable and accessible format?

Yes

Are the protocols appropriate and is the work technically sound?

Yes

Reviewer Expertise:

Ecology - Evolution - Phylogenetics - Systematics

I confirm that I have read this submission and believe that I have an appropriate level of expertise to confirm that it is of an acceptable scientific standard.

# Reviewer report: Reviewer response for version 1

**DOI:** 10.21956/openreseurope.24731.r69973

**Published:** 2026-03-10

**Authors:** Javier Blasco-Aróstegui

**Affiliations:** 1 Universidade de Lisboa, Lisbon, Lisbon, Portugal

The article provides a reference chromosome-level genome for the troglomorphic scorpion *Belisarius xambeui*, endemic to France and Spain, and belonging to an incertae sedis lineage within the scorpion Tree of Life. This work has the potential to shed light on the evolution of this group and help clarify its phylogenetic position within the Scorpion Tree of Life. I believe this contribution is timely and appropriate, and I therefore recommend the article for indexing in Open Research Europe.

 However, several minor edits and comments should be addressed prior to acceptance. These are important to clarify certain aspects of the manuscript and to ensure that relevant information is presented accurately.

**Title:** It is arguably *“the most singular scorpion in Europe”*, considering that there are supposedly two species in the genus *Belisarius*, and the closely related species *Sardoscorpius troglophilus* might be even more “singular” due to its rarity. I would recommend avoiding this second fragment of the title. 

**“Bauplan”**

 Consider replacing this term with “body plan,” which is more widely used and readily understood in the literature.

**“Stiff bristles”**

 I am not certain that this represents a diagnostic character for this taxon. Please provide an appropriate reference if this feature is indeed considered diagnostic. Moreover, European euscorpiids also possess bristles on the chelicerae. Other characters may be more informative for defining this lineage of scorpions, such as the trichobothrial pattern, which is typically regarded as more diagnostic.

**“it is considered the most cryptic in Europe”**

 This appears to be a rather strong statement that is supported only by an older reference (Vignoli et al., 2008). Some populations of its congener *B. ibericus* appear to be even more relictual or cryptic, as they have not been rediscovered since their original description to the best of my knowledge. A similar case could be made for *S. troglophilus*, which has never been recaptured and is known only from the original material. Other European examples include the troglobitic *Euscorpius studentium *from Montenegro, which is known from only two adult specimens and a few juveniles. The authors may wish to soften this statement or provide additional support.

**“Moreover, its low fertility (5–24 eggs per clutch) may further compromise its long-term survival”**

 Please provide a reference supporting this statement.

**“It was initially allied with the family Troglotayosicidae, which comprises a single genus of cave-dwelling species native to Ecuador and Colombia”**

 This statement requires clarification. The genus *Troglotayosicus* largely comprises humicolous species inhabiting leaf litter, and only one species ( *Troglotayosicus vachoni*) is truly cave-dwelling.

**“the new family Belisariidae has been created to include the blind scorpion along with a recently described cave-dwelling species from southern Iberian Peninsula ( *B. ibericus* Lourenço, 2015)”**

 Please note that the family Belisariidae was originally described by Lourenço (1998). It was only recently resurrected to accommodate the species mentioned here. The wording should be adjusted to reflect this taxonomic history.

**“At present, genomic data are available for only six scorpion species”**

 In a brief search, I found that a chromosome-level genome assembly is also available for *Olivierus przewalskii (= Mesobuthus przewalskii*; Su et al., 2025). The authors may wish to include this reference and, ideally, cite the other available scorpion genomes as well.

**“Sex of the individuals collected is unknown”**

 It should be possible to determine the sex of the individuals used for the genome assembly by examining the number and morphology of the pectinal teeth, as well as the genital operculum. The literature on *Belisarius* should provide information on the typical counts for males and females. See for example:

 Refer to reference 1&2 

**Individuals from different localities**

 According to the information available, *Belisarius xambeui* populations appear to represent a clear example of a genetic bottleneck. It may therefore be important to justify this in the methodology as to why individuals from different localities were used rather than specimens from the same population, as otherwise this could have potentially influenced the genomic analyses.

**Figure 1**

 A more detailed map would be appropriate for the article. The current map lacks the sampling localities used in the study; they should be in a different color or shape. It may also be useful to include an elevation layer, which would help illustrate the altitudinal gradient across the distribution of the species. Additionally, if occurrence data were obtained from GBIF, the authors should ensure that records corresponding to museum coordinates rather than actual collection localities were filtered out.

**References**

 The authors may also wish to cite several relevant works on the taxa in question, including:(Refer to reference 3, 4, & 5)

Are sufficient details of methods and materials provided to allow replication by others?

Partly

Is the rationale for creating the dataset(s) clearly described?

Yes

Are the datasets clearly presented in a useable and accessible format?

Yes

Are the protocols appropriate and is the work technically sound?

Yes

Reviewer Expertise:

Ecology - Evolution - Phylogenetics - Systematics

I confirm that I have read this submission and believe that I have an appropriate level of expertise to confirm that it is of an acceptable scientific standard.

## References

[1] : Descrizione del maschio del Belisarius xambeui E. Sim. (Arachnida, Scorpiones) de la Catalogna settentrionale. *Publ. Junta Cien. Nat. Barcelona 4: 1–6* .1924;

[2] : Remarques sur le scorpion aveugle du Roussillon: Belisarius xambeui E. Simon. *Bull. Mus. Natl. Hist. Nat. 16: 298–305* .1945;

[3] : 3e Ordre – Scorpiones. In: Les Arachnides de France. VII. Contenant les Ordres des Chernetes, Scorpiones et Opiliones. *Roret, Paris, pp. 79–115* .1879;

[4] : The genus Belisarius Simon, 1879 (Scorpiones: Troglotayosicidae), with the description of a new vicariant species from southern Spain. *Comptes Rendus Biologies, 338: 362–367* .2015;10.1016/j.crvi.2015.03.01425898979

[5] : A remarkable discovery of a new scorpion genus and species from Sardinia (Scorpiones: Chactoidea: Belisariidae). *Arachnida – Rivista Aracnologica Italiana, 26: 3–25* .2020;

# Response

**Published:** 2026-03-20

**Authors:** Julio Rozas

**Affiliations:** Departament de Genetica Microbiologia i Estadistic, Universitat de Barcelona Departament de Genetica Microbiologia i Estadistica, Barcelona, Catalonia, Spain

The article provides a reference chromosome-level genome for the troglomorphic scorpion Belisarius xambeui, endemic to France and Spain, and belonging to an incertae sedis lineage within the scorpion Tree of Life. This work has the potential to shed light on the evolution of this group and help clarify its phylogenetic position within the Scorpion Tree of Life. I believe this contribution is timely and appropriate, and I therefore recommend the article for indexing in Open Research Europe.

 However, several minor edits and comments should be addressed prior to acceptance. These are important to clarify certain aspects of the manuscript and to ensure that relevant information is presented accurately.

 Title: It is arguably “the most singular scorpion in Europe”, considering that there are supposedly two species in the genus Belisarius, and the closely related species Sardoscorpius troglophilus might be even more “singular” due to its rarity. I would recommend avoiding this second fragment of the title. 

**We agree, we decided to modify this second fragment of the title to: … a singular scorpion in Europe.**

 “Bauplan”

 Consider replacing this term with “body plan,” which is more widely used and readily understood in the literature.

**We replaced the term as suggested.**

 “Stiff bristles”

 I am not certain that this represents a diagnostic character for this taxon. Please provide an appropriate reference if this feature is indeed considered diagnostic. Moreover, European euscorpiids also possess bristles on the chelicerae. Other characters may be more informative for defining this lineage of scorpions, such as the trichobothrial pattern, which is typically regarded as more diagnostic.

**We agree. Thus, we have removed this part in the new versión of the ms.**

 “it is considered the most cryptic in Europe”

 This appears to be a rather strong statement that is supported only by an older reference (Vignoli et al., 2008). Some populations of its congener B. ibericus appear to be even more relictual or cryptic, as they have not been rediscovered since their original description to the best of my knowledge. A similar case could be made for S. troglophilus, which has never been recaptured and is known only from the original material. Other European examples include the troglobitic Euscorpius studentium from Montenegro, which is known from only two adult specimens and a few juveniles. The authors may wish to soften this statement or provide additional support.

**We agree, we have modified this sentence in the new versión of the ms.**

 “Moreover, its low fertility (5–24 eggs per clutch) may further compromise its long-term survival”

 Please provide a reference supporting this statement.

**We added Auber 1959 as a reference to support this statement.**

 “It was initially allied with the family Troglotayosicidae, which comprises a single genus of cave-dwelling species native to Ecuador and Colombia”

 This statement requires clarification. The genus Troglotayosicus largely comprises humicolous species inhabiting leaf litter, and only one species (Troglotayosicus vachoni) is truly cave-dwelling.

**We thank the reviewer for the clarification. We added in the text that this genus also comprises humicolous species.**

 “the new family Belisariidae has been created to include the blind scorpion along with a recently described cave-dwelling species from southern Iberian Peninsula (B. ibericus Lourenço, 2015)”

 Please note that the family Belisariidae was originally described by Lourenço (1998). It was only recently resurrected to accommodate the species mentioned here. The wording should be adjusted to reflect this taxonomic history.

**We agree with the reviewer and corrected the sentence in the text as suggested.**

 “At present, genomic data are available for only six scorpion species”

 In a brief search, I found that a chromosome-level genome assembly is also available for Olivierus przewalskii (= Mesobuthus przewalskii; Su et al., 2025). The authors may wish to include this reference and, ideally, cite the other available scorpion genomes as well.

 Added  

 “Sex of the individuals collected is unknown”

 It should be possible to determine the sex of the individuals used for the genome assembly by examining the number and morphology of the pectinal teeth, as well as the genital operculum. The literature on Belisarius should provide information on the typical counts for males and females. See for example:

 Refer to reference 1&2 

**We thank the reviewer for the information and references. Unfortunately, a careful and detailed examination was not possible since the specimens needed to be flash frozen in order to ensure an optimal preservation for high molecular weight DNA extraction.**

  Individuals from different localities

 According to the information available, Belisarius xambeui populations appear to represent a clear example of a genetic bottleneck. It may therefore be important to justify this in the methodology as to why individuals from different localities were used rather than specimens from the same population, as otherwise this could have potentially influenced the genomic analyses.

**We agree with the reviewer. However, because specimens of *Belisarius xambeui* are rare, and in order to secure the highest quality of DNA, we used specimens in the best condition of preservation, which motivated the decision to use specimens from different localities.**

 Figure 1

 A more detailed map would be appropriate for the article. The current map lacks the sampling localities used in the study; they should be in a different color or shape. It may also be useful to include an elevation layer, which would help illustrate the altitudinal gradient across the distribution of the species. Additionally, if occurrence data were obtained from GBIF, the authors should ensure that records corresponding to museum coordinates rather than actual collection localities were filtered out.

**We have included a new versión of this figure in the new versión of the ms.**

****References

 The authors may also wish to cite several relevant works on the taxa in question, including:(Refer to reference 3, 4, & 5)

**Added**

# Reviewer report: Reviewer response for version 1

**DOI:** 10.21956/openreseurope.24731.r69968

**Published:** 2026-03-04

**Authors:** Carlos Fernando Prada Quiroga

**Affiliations:** 1 University of Tolima, Ibague, Colombia

The manuscript entitled *“ERGA-BGE-CBP chromosome-level genome assembly of the blind scorpion* Belisarius xambeui *Simon, 1879 (Belisariidae, Scorpiones), the most singular scorpion in Europe”* presents the sequencing and chromosome-level assembly of the genome of this species. The study is generally well structured and adequately supported.

 However, several methodological aspects require clarification and additional detail before the manuscript can be considered suitable for indexing.

 1. Sample Collection, Processing, and Genome Size Estimation:

 a) The section would benefit from a more detailed description of the taxonomic identification process. Please specify how the individuals were identified (e.g., diagnostic morphological characters, taxonomic keys, expert validation). Additionally, indicate whether voucher specimens were deposited in a recognized biological collection, including accession numbers if available. This information is essential to ensure traceability and reproducibility.

 b) The authors state that “the sex of these individuals is unknown.” Although sexual dimorphism in this taxon is minimal, it is important to clarify that sex determination may still be feasible through detailed examination of the genital operculum. While there are no obvious external morphological differences between males and females, sex identification can be achieved through careful analysis of genital structures (see, for example, Lourenço, 2015). The authors should clarify whether such examination was attempted and, if not, justify its omission.

 2. Sampling Localities for PacBio and Hi-C

 The manuscript indicates that one individual from Cova de la Mosquera (Beuda, Girona) was used for PacBio sequencing, while a third individual from Cantonigròs (Santa Maria de Corcó) was used for Hi-C scaffolding. The rationale for selecting individuals from different localities should be explicitly justified.

 Using specimens from distinct populations may introduce structural variation that could potentially affect genome scaffolding and assembly accuracy. Please clarify whether population-level genomic variation was considered and how potential structural differences were controlled for or evaluated.

 3. Figure Quality

 Figure 2C appears to be of moderate to low resolution. It is recommended that the authors replace this image with a higher-quality photograph in which diagnostic morphological features of the organism are more clearly visible.

 4. DNA Extraction and Quantification

 In the DNA extraction section, please provide detailed information on DNA quantification procedures, including:

 The method used for quantification (e.g., fluorometric, spectrophotometric).

 The equipment and kits employed.

 Quality assessment metrics (e.g., A260/280, A260/230 ratios, fragment size evaluation).Including this information will improve methodological transparency and reproducibility.

 5. Genome Annotation

 More importantly, the authors should provide a clear justification for not performing gene annotation on the assembled genome. Even in draft assemblies, structural and functional annotation is generally considered a fundamental component of genome characterization. If annotation was not conducted due to assembly limitations (e.g., fragmentation, incomplete scaffolding) or other constraints, this should be explicitly stated and discussed. Otherwise, the absence of gene annotation significantly limits the biological interpretation and utility of the genomic resource

Are sufficient details of methods and materials provided to allow replication by others?

Partly

Is the rationale for creating the dataset(s) clearly described?

Yes

Are the datasets clearly presented in a useable and accessible format?

Yes

Are the protocols appropriate and is the work technically sound?

Yes

Reviewer Expertise:

NA

I confirm that I have read this submission and believe that I have an appropriate level of expertise to confirm that it is of an acceptable scientific standard, however I have significant reservations, as outlined above.

# Response

**Published:** 2026-03-18

**Authors:** Julio Rozas

**Affiliations:** Departament de Genetica Microbiologia i Estadistic, Universitat de Barcelona Departament de Genetica Microbiologia i Estadistica, Barcelona, Catalonia, Spain

1a). Sample Collection, Processing, and Genome Size Estimation:

 a) The section would benefit from a more detailed description of the taxonomic identification process. Please specify how the individuals were identified (e.g., diagnostic morphological characters, taxonomic keys, expert validation). Additionally, indicate whether voucher specimens were deposited in a recognized biological collection, including accession numbers if available. This information is essential to ensure traceability and reproducibility.

 We agree. We have added a sentence deling with that in the new versión of the ms.   1b) The authors state that “the sex of these individuals is unknown.” Although sexual dimorphism in this taxon is minimal, it is important to clarify that sex determination may still be feasible through detailed examination of the genital operculum. While there are no obvious external morphological differences between males and females, sex identification can be achieved through careful analysis of genital structures (see, for example, Lourenço, 2015). The authors should clarify whether such examination was attempted and, if not, justify its omission. 

 We have added a sentence deling with that in the new versión of the ms. 

 2. Sampling Localities for PacBio and Hi-C

 The manuscript indicates that one individual from Cova de la Mosquera (Beuda, Girona) was used for PacBio sequencing, while a third individual from Cantonigròs (Santa Maria de Corcó) was used for Hi-C scaffolding. The rationale for selecting individuals from different localities should be explicitly justified.

 Using specimens from distinct populations may introduce structural variation that could potentially affect genome scaffolding and assembly accuracy. Please clarify whether population-level genomic variation was considered and how potential structural differences were controlled for or evaluated.

 We have justified the reasons of our samplins strategy in the new versión of the ms. 

 3. Figure Quality

 Figure 2C appears to be of moderate to low resolution. It is recommended that the authors replace this image with a higher-quality photograph in which diagnostic morphological features of the organism are more clearly visible.

   We have included a new versión of this figure in the new versión of the ms.   

 4. DNA Extraction and Quantification

 In the DNA extraction section, please provide detailed information on DNA quantification procedures, including:

 The method used for quantification (e.g., fluorometric, spectrophotometric).

 The equipment and kits employed.

 Quality assessment metrics (e.g., A260/280, A260/230 ratios, fragment size evaluation).Including this information will improve methodological transparency and reproducibility.

   We have included this information in the new versión of the ms. We measured the DNA extraction quality uisng a NanoDrop spectrophotomer, yielding absorbance ratios of  260/280 = 1.74  and  260/230 =  2.26 

 5. Genome Annotation

 More importantly, the authors should provide a clear justification for not performing gene annotation on the assembled genome. Even in draft assemblies, structural and functional annotation is generally considered a fundamental component of genome characterization. If annotation was not conducted due to assembly limitations (e.g., fragmentation, incomplete scaffolding) or other constraints, this should be explicitly stated and discussed. Otherwise, the absence of gene annotation significantly limits the biological interpretation and utility of the genomic resource We completly agree. However, we have not yet finished this bioinformatic task. Once completed we will include the information in the Belisarius repository on GitHub (indicated in the ms).

# Reviewer report: Reviewer response for version 1

**DOI:** 10.21956/openreseurope.24731.r69972

**Published:** 2026-03-03

**Authors:** Carlos E. Santibáñez-López

**Affiliations:** 1 Western Connecticut State University, Danbury, Connecticut, USA

This manuscript represents a significant contribution to the field of arachnid genomics. The authors have provided a robust framework for understanding scorpion genome architecture, and the methodology is both sound and transparent. The commitment to data sharing is commendable. I recommend this manuscript for indexing pending the following minor revisions to ensure the literature and divergence estimates are fully up to date.

 The authors discuss the unique life history of *Belisarius xambeui*; however, the claim regarding the **low fertility** of this species requires a formal citation. Please include the appropriate primary literature to support this observation.

 The manuscript notes that there are currently six published scorpion genomes. However, the authors should incorporate the recently published genome of ***Olivierus przewalskii*** (= *Mesobuthus przewalskii*; Su et al., 2025).

 The divergence estimates provided for the split between **Buthida** and **Iurida** should be updated to reflect recent large-scale phylogenomic analyses. I recommend using the estimate of **291–320 Ma (Carboniferous–Permian)** as recovered by the comprehensive analysis in Santibáñez-López et al. (2022).

Are sufficient details of methods and materials provided to allow replication by others?

Yes

Is the rationale for creating the dataset(s) clearly described?

Yes

Are the datasets clearly presented in a useable and accessible format?

Yes

Are the protocols appropriate and is the work technically sound?

Yes

Reviewer Expertise:

Arachnid Evolution - Genomics - Venomics

I confirm that I have read this submission and believe that I have an appropriate level of expertise to confirm that it is of an acceptable scientific standard.

## References

[1] Phylogenomics of scorpions reveal contemporaneous diversification of scorpion mammalian predators and mammal-active sodium channel toxins. Systematic Biology, 71(6), 1281-1289. 10.1093/sysbio/syac02135348798

[2] A chromosome-level genome assembly for the desert scorpion Mesobuthus przewalskii from Asian drylands. Journal of Heredity,.10.1093/jhered/esae05939432580

# Response

**Published:** 2026-03-20

**Authors:** Julio Rozas

**Affiliations:** Departament de Genetica Microbiologia i Estadistic, Universitat de Barcelona Departament de Genetica Microbiologia i Estadistica, Barcelona, Catalonia, Spain

This manuscript represents a significant contribution to the field of arachnid genomics. The authors have provided a robust framework for understanding scorpion genome architecture, and the methodology is both sound and transparent. The commitment to data sharing is commendable. I recommend this manuscript for indexing pending the following minor revisions to ensure the literature and divergence estimates are fully up to date.

 1. The authors discuss the unique life history of Belisarius xambeui; however, the claim regarding the low fertility of this species requires a formal citation. Please include the appropriate primary literature to support this observation.

**We have added a sentence dealing with that in the new version of the ms.**

 2. The manuscript notes that there are currently six published scorpion genomes. However, the authors should incorporate the recently published genome of Olivierus przewalskii (= Mesobuthus przewalskii; Su et al., 2025).

**We have modified this sentence in the new version of the ms.**

 3. The divergence estimates provided for the split between Buthida and Iurida should be updated to reflect recent large-scale phylogenomic analyses. I recommend using the estimate of 291–320 Ma (Carboniferous–Permian) as recovered by the comprehensive analysis in Santibáñez-López et al. (2022).  

**We agree. We have modified this sentence in the new version of the ms.**

# Reviewer report: Reviewer response for version 1

**DOI:** 10.21956/openreseurope.24731.r69975

**Published:** 2026-02-27

**Authors:** Cheng-Min Shi

**Affiliations:** 1 Hebei Agricultural University, Baoding, China

This data note reports a genome assembly for Belisarius *xambeui,  *a scorpion species that is endemic to Catalan. *Belisarius xambeui *belongs to the species-poor family Belisariidae, which represents a unique lineage of scorpions as signified by troglomorphism.  The assembly is an important contribution to the genome resources not only to scorpions but also to troglomorphic arthropods.  The genome reported is the largest one of the sequenced genomes of scorpions to date.  In general, the manuscript is well written. I only have the following remarks.

 1. The last sentence of the Abstract should be better expressed as ''The average nucleotide diversity *π* across the genome is 0.0018 (range 0.0011~0.0028)'.  Without a reference species or population, it is not prudent to say the diversity is low or not.

 2.  Background--- At present, genomic data are available for at least 7 scorpion species.  See Su et al. 2024. Journal of Heredity 116(4): 532-539 for a chromosome-level genome assembly for *Mesobuthus przewalskii* (Buthidae).

 3. Data availability ---accessions for mitochondrial genome and HiC data should be provided.

Are sufficient details of methods and materials provided to allow replication by others?

Yes

Is the rationale for creating the dataset(s) clearly described?

Partly

Are the datasets clearly presented in a useable and accessible format?

Yes

Are the protocols appropriate and is the work technically sound?

Yes

Reviewer Expertise:

Scorpion molecular ecology and evolutionary genomics

I confirm that I have read this submission and believe that I have an appropriate level of expertise to confirm that it is of an acceptable scientific standard.

# Response

**Published:** 2026-03-18

**Authors:** Julio Rozas

**Affiliations:** Departament de Genetica Microbiologia i Estadistic, Universitat de Barcelona Departament de Genetica Microbiologia i Estadistica, Barcelona, Catalonia, Spain

1. The last sentence of the Abstract should be better expressed as ''The average nucleotide diversity π across the genome is 0.0018 (range 0.0011~0.0028)'.  Without a reference species or population, it is not prudent to say the diversity is low or not. We agree. We have added a sentence deling with that in the new versión of the ms. 

 2.  Background--- At present, genomic data are available for at least 7 scorpion species.  See Su et al. 2024. Journal of Heredity 116(4): 532-539 for a chromosome-level genome assembly for Mesobuthus przewalskii (Buthidae). Added 

 3. Data availability ---accessions for mitochondrial genome and HiC data should be provided.  Absolutely. The accession numbers have already been included.

# Response

**Published:** 2026-03-19

**Authors:** Julio Rozas

**Affiliations:** Departament de Genetica Microbiologia i Estadistic, Universitat de Barcelona Departament de Genetica Microbiologia i Estadistica, Barcelona, Catalonia, Spain

1. The last sentence of the Abstract should be better expressed as ''The average nucleotide diversity π across the genome is 0.0018 (range 0.0011~0.0028)'.  Without a reference species or population, it is not prudent to say the diversity is low or not. We agree. We have added a sentence deling with that in the new versión of the ms.  

 2.  Background--- At present, genomic data are available for at least 7 scorpion species.  See Su et al. 2024. Journal of Heredity 116(4): 532-539 for a chromosome-level genome assembly for Mesobuthus przewalskii (Buthidae). Added  

 3. Data availability ---accessions for mitochondrial genome and HiC data should be provided.  Absolutely. The accession numbers have already been included.
